# Progress Toward Measles Elimination — Eastern Mediterranean Region, 2013–2019

**DOI:** 10.15585/mmwr.mm6915a2

**Published:** 2020-04-17

**Authors:** James L. Goodson, Nadia Teleb, Hossam Ashmony, Nasrin Musa, Amany Ghoniem, Quamrul Hassan, Abdul Shakoor Waciqi, Mohammed Osama Mere, Muhammad Farid, Hanan Elhag Abdo Mukhtar, Javed Iqbal, James P. Alexander

**Affiliations:** ^1^Global Immunization Division, Center for Global Health, CDC; ^2^Vaccine Preventable Diseases and Immunization, World Health Organization Eastern Mediterranean Regional Office, Egypt; ^3^Expanded Programme on Immunization, World Health Organization Country Office, Afghanistan; ^4^Expanded Programme on Immunization, World Health Organization Country Office, Pakistan; ^5^Expanded Programme on Immunization, World Health Organization Country Office, Somalia; ^6^Expanded Programme on Immunization, World Health Organization Country Office, Sudan; ^7^Expanded Programme on Immunization, World Health Organization Country Office, Yemen.

In 1997, during the 41st session of the Regional Committee for the Eastern Mediterranean, the 21 countries in the World Health Organization (WHO) Eastern Mediterranean Region* (EMR) passed a resolution to eliminate^†^ measles (*1*). In 2015, this goal was included as a priority in the Eastern Mediterranean Vaccine Action Plan 2016–2020 (EMVAP) (*2*), endorsed at the 62nd session of the Regional Committee (*3*). To achieve this goal, the WHO Regional Office for the Eastern Mediterranean developed a four-pronged strategy: 1) achieve ≥95% vaccination coverage with the first dose of measles-containing vaccine (MCV1) among children in every district of each country through routine immunization services; 2) achieve ≥95% vaccination coverage with a second MCV dose (MCV2) in every district of each country either through implementation of a routine 2-dose vaccination schedule or through supplementary immunization activities^§^ (SIAs); 3) conduct high-quality, case-based surveillance in all countries; and 4) provide optimal measles clinical case management, including dietary supplementation with vitamin A (*4*). This report describes progress toward measles elimination in EMR during 2013–2019 and updates a previous report (*5*). Estimated MCV1 coverage increased from 79% in 2013 to 82% in 2018. MCV2 coverage increased from 59% in 2013 to 74% in 2018. In addition, during 2013–2019, approximately 326.4 million children received MCV during SIAs. Reported confirmed measles incidence increased from 33.5 per 1 million persons in 2013 to 91.2 in 2018, with large outbreaks occurring in Pakistan, Somalia, and Yemen; incidence decreased to 23.3 in 2019. In 2019, the rate of discarded nonmeasles cases^¶^ was 5.4 per 100,000 population. To achieve measles elimination in the EMR, increased visibility of efforts to achieve the measles elimination goal is critically needed, as are sustained and predictable investments to increase MCV1 and MCV2 coverage, conduct high-quality SIAs, and reach populations at risk for not accessing immunization services or living in areas with civil strife.

## Immunization Activities

MCV1 and MCV2 administrative coverage** data are reported each year from all EMR countries and areas to WHO and the United Nations Children’s Fund (UNICEF) through the Joint Reporting Form. WHO and UNICEF use reported administrative coverage and available survey results to generate annual estimates of vaccination coverage through routine immunization services (*6*). During 2013–2018, estimated regional MCV1 coverage increased from 79% to 82%, and estimated MCV2 coverage increased from 59% to 74% (Table 1). In 2018, 11 (52%) of 21 countries and areas achieved ≥95% coverage with both MCV1 and MCV2. As of 2018, only one (5%) EMR country (Somalia) had not yet introduced MCV2. During 2013–2019, 326.4 million persons were vaccinated during 89 SIAs, with weighted regional SIA coverage of 98% (Table 2). Reported vaccination coverage was ≥90% in 25 (68%) of 37 nationwide SIAs, including ≥95% in 11 (30%).

**TABLE 1:** Measles-containing vaccine (MCV) schedule, estimated coverage with the first and second doses of MCV,* number of confirmed measles cases,^†^ and confirmed measles incidence, by country/area — World Health Organization (WHO) Eastern Mediterranean Region, 2013, 2018, and 2019

Country/Area	MCV schedule^§^		2013				2018				2019^¶^	
			Coverage (%)		No. of measles cases	Incidence**	Coverage (%)		No. of measles cases	Incidence**	No. of measles cases	Incidence**
	Age–1st dose (mos)	Age–2nd dose (mos)	MCV1	MCV2			MCV1	MCV2				
Afghanistan	9	18	57	35	430	13.3	64	39	2,012	54.1	183	4.8
Bahrain	12	18	99	99	0	0.0	99	99	0	0.0	0	0.0
Djibouti	9	15	80	82	28	31.7	86	81	28	29.2	NR	NR
Egypt	12	18	96	96	405	4.6	94	94	23	0.2	0	0.0
Iran	12	18	98	97	189	2.5	99	98	203	2.5	0	0.0
Iraq	9	15	72	57	669	20.2	83	81	489	12.7	721	18.3
Jordan^††^	12	18	97	98	120	14.1	92	96	0	0.0	45	4.5
Kuwait^§§^	12	24	99	99	62	17.6	99	99	34	8.2	12	2.9
Lebanon^††^	12	18	82	65	1,761	297.8	82	63	943	137.5	1,069	155.9
Libya	12	18	96	95	164	25.9	97	96	1,059	158.6	188	27.7
Morocco	9	18	99	NA^§§^	92	2.7	99	99	8	0.2	12	0.3
Oman	12	18	99	99	0	0.0	99	99	0	0.0	0	0.0
Pakistan	9	15	68	43	8,749	45.7	76	67	33,007	155.5	2,066	9.5
Palestine	12	18	99	98	0	0.0	99	99	0	0.0	163	32.7
Qatar	12	18	97	99	73	31.2	99	95	2	0.7	5	1.8
Saudi Arabia^††,§§^	12	18	98	99	1,164	38.7	98	97	1,161	34.4	956	27.9
Somalia	9	NA^¶¶^	46	NA^¶¶^	3,173	242.9	46	NA^¶¶^	9,124	607.9	4,482	290.2
Sudan	9	18	86	57	2,813	75.9	88	72	4,980	119.1	3,555	83.0
Syria	12	18	58	51	740	37.8	63	54	329	19.4	27	1.6
Tunisia	12	18	94	98	16	1.5	96	99	12	1.0	1,870	159.9
United Arab Emirates^§§^	12	18	98	98	309	33.6	99	99	172	17.9	186	19.0
Yemen	9	18	70	47	400	15.9	64	46	10,640	373.4	1,163	39.9
**EMR**	**—**	—	**79**	**59**	**21,357**	**33.5**	**82**	**74**	**64,226**	**91.2**	**16,703**	**23.3**

**Abbreviations:** EMR = Eastern Mediterranean Region; MCV = measles-containing vaccine; MCV1 = first MCV dose; MCV2 = second MCV dose; NA = not applicable; NR = not reported.

* WHO and United Nations Children's Fund Estimates of National Immunization Coverage (WUENIC). For MCV1, among children aged 1 year or, if MCV1 is given at age ≥1 year, among children aged 24 months. For MCV2, among children at the recommended age for administration of MCV2, per the national immunization schedule. The WUENIC were last revised on July 15, 2019, and are available at https://www.who.int/immunization/monitoring_surveillance/data/en.

^†^ Includes cases confirmed by laboratory or epidemiologic linkage and clinically compatible cases. Clinically compatible cases met the WHO measles clinical case definition, had no adequate specimen collected, and could not be epidemiologically linked to a laboratory-confirmed case of measles.

^§^ MCV schedule is the 2019 schedule.

^¶^ 2019 MCV1 and MCV2 coverage estimates not available at time of publication.

** Cases per million population.

^††^ Additional 9-month dose provided nationally.

^§§^ Additional dose provided nationally at age 5–6 years (United Arab Emirates), 6 years (Saudi Arabia), or 12 years (Kuwait).

^¶¶^ Dose was not included in the vaccination schedule for that year.

**TABLE 2:** Characteristics of measles supplementary immunization activities (SIAs),* by year and country/area — World Health Organization Eastern Mediterranean Region, 2013–2019

Year	Country/Area	Age group targeted^†^	Measles-containing vaccine used	Extent of SIA	Population reached in targeted age group, no. (%)
2013	Afghanistan	9m–59m	M	Subnational	875,874 (85)
	Iran	9m–12y	MMR	Subnational	157,000 (97)
	Iraq	6y–12y	M	National	5,563,532 (96)
	Jordan	9m–14y	M	Subnational	639,420 (>100)
	Jordan	9m–14y	MR	National	3,361,516 (>100)
	Lebanon	9m–18y	M	Subnational	294,079 (85)
	Lebanon	9m–18y	M	Subnational	308,438 (76)
	Morocco	9m–19y	MR	National	10,191,571 (91)
	Pakistan	9m–9y	M	Subnational	4,002,154 (>100)
	Pakistan	6m–9y	M	Subnational	26,986,015 (96)
	Somalia	9m–59m	M	Subnational	923,580 (90)
	Sudan	9m–15y	M	National	14,976,050 (98)
	Syria	6y–10y	MMR	National	789,678 (72)
	Syria	12y–15y	MMR	National	759,427 (92)
	Yemen	6m–10y	M	Subnational	283,687 (93)
2014	Afghanistan	6m–10y	M	Subnational	321,750 (92)
	Afghanistan	9m–59m	M	Subnational	520,384 (95)
	Iraq	9m–59m	M	National	3,295,122 (96)
	Lebanon	9m–18y	MR	National	1,056,830 (72)
	Pakistan	6m–9y	M	Subnational	9,432,492 (>100)
	Pakistan	6m–9 y	M	Subnational	14,026,013 (>100)
	Pakistan	6m–9y	M	Subnational	1,439,892 (100)
	Somalia	9m–59m	M	Subnational	1,306,426 (88)
	Syria	7m–5y	MMR	National	766,305 (74)
	Yemen	9m–15y	MR	National	11,368,968 (93)
2015	Afghanistan	9–59m	M	National	6,191,955 (>100)
	Djibouti	9m–15y	M	National	277,119 (91)
	Djibouti	15y–25y	M	National	169,493 (76)
	Egypt	9m–10y	MR	National	23,356,156 (>100)
	Iran	9m–15y	MR	Subnational	1,804,000 (99)
	Iraq	9m–5y	MR	National	4,499,656 (94)
	Pakistan	6m–10y	M	Subnational	30,633,406 (>100)
	Pakistan	6m–10y	M	Subnational	227,762 (95)
	Pakistan	6m–10y	M	Subnational	204,308 (>100)
	Pakistan	6m–10y	M	Subnational	3,512,771 (>100)
	Pakistan	6m–10y	M	Subnational	413,695 (100)
	Pakistan	6m–10y	M	Subnational	1,519,242 (95)
	Somalia	9m–9y	M	Subnational	3,518,358 (91)
	Sudan	6m–15y	M	Subnational	1,026,990 (96)
	Sudan	6m–15y	M	Subnational	1,716,997 (>100)
	Sudan	6m–15y	M	Subnational	3,541,601 (100)
	Sudan	6m–15y	M	Subnational	3,078,800 (>100)
	Syria	6m–59m	MMR	National	1,619,630 (61)
	United Arab Emirates	1y–18y	MMR	National	915,480 (69)
	Yemen	6m–15y	MR	Subnational	1,590,462 (85)
2016	Afghanistan	9m–10y	M	Subnational	2,450,393 (>100)
	Egypt	11y–20y	MR	Subnational	642,178 (94)
	Egypt	6y	MR	Subnational	258,464 (>100)
	Iraq	6y	MMR	Subnational	722,680 (>100)
	Qatar	1y–13y	MMR	National	166,145 (87)
	Somalia	9m–59m	M	National	602,136 (89)
	Somalia	9m–59m	M	Subnational	140,533 (74)
	Sudan	6m–15y	M	Subnational	4,383,506 (>100)
	Syria	9m–59m	MR	Subnational	927,820 (91)
	United Arab Emirates	19y–34y	MMR	National	581,519 (46)
	Yemen	6m–15y	MR	Subnational	2,421,243 (92)
2017	Afghanistan	9–59m	M	Subnational	1,053,452 (97)
	Djibouti	4y–8y	M	National	11,628 (92)
	Iraq	6y–13y	MMR	Subnational	319,314 (82)
	Kuwait	1y–19y	MMR	National	165,296 (16)
	Libya	3y–6y	MMR	National	721,488 (>100)
	Oman	20y–35y	MMR	National	1,658,642 (92)
	Pakistan	9m–59m	M	Subnational	1,279,819 (94)
	Somalia	6m–59m	M	National	4,400,000 (94)
	Somalia	6m–59m	M	Subnational	472,033 (94)
	Sudan	9m–15y	M	Subnational	2,066,281 (>100)
	Sudan	6m–>15y**^§^**	M	Subnational	73,680 (98)
	Syria	7m–59m	M	National	1,779,459 (72)
	Syria	5y–12y	MMR	National	2,978,998 (82)
	Yemen	6m–15y	MR	Subnational	205,731 (41)
	Yemen	6m–15y	MR	Subnational	166,654 (100)
2018	Afghanistan	9m–10y	M	National	12,590,923 (91)
	Djibouti	6m–59m	M	National	113,780 (>100)
	Iraq	9m–5y	MMR	National	2,095,740 (93)
	Libya	9m–14y	MR	National	2,654,466 (96)
	Pakistan	9m–10y	M	Subnational	91,111 (99)
	Pakistan	6m–59m	M	Subnational	914,058 (87)
	Pakistan	9m–59m	M	National	37,131,234 (>100)
	Somalia	6m–10y	M	National	4,496,540 (93)
	Syria	6y–12y	M	Subnational	1,439,848 (99)
	Syria	6y–12y	M	Subnational	1,142,817 (86)
	Yemen	6m–10y	MR	Subnational	572,961 (85)
	Yemen	6m–15y	MR	Subnational	294,452 (74)
2019	Iraq	9m–59m	MMR	National	2,421,421 (90)
	Jordan	6m–6y	M	Subnational	81,576 (90)
	Lebanon	6m–10y	M and MMR	National	253,204 (82)
	Somalia	6m–59m	M	Subnational	1,051,504 (91)
	Sudan	9m–15y	M	National	13,027,696 (98)
	Yemen	6m–14y	MR	National	11,959,569 (93)
**2013–2019**	**EMR**	**—**	**—**	**—**	**326,446,076 (98**)^¶^

**Abbreviations:** EMR = Eastern Mediterranean Region; M = measles vaccine; MMR = measles, mumps, and rubella vaccine; MR = measles and rubella vaccine.

*SIAs generally are carried out using two approaches. An initial, nationwide catch-up SIA targets all children aged 9 months–14 years; it has the goal of eliminating susceptibility to measles in the general population. Periodic follow-up SIAs then target all children born since the last SIA. Follow-up SIAs generally are conducted nationwide every 2–4 years and generally target children aged 9–59 months; their goal is to eliminate any measles susceptibility that has developed in recent birth cohorts and to protect children who did not respond to the first measles vaccination. The exact age range for follow-up SIAs depends on the age-specific incidence of measles, coverage with measles-containing vaccine through routine services, and the time since the last SIA.

**^†^** Targeted age groups varied by province.

^§^ Outbreak response immunization campaign that targeted children aged 6 months through 15 years and also young miners aged ≥15 years.

^¶^ Average SIA coverage, weighted by size of target population.

## Surveillance Activities

Case-based measles surveillance^††^ data are reported monthly to WHO from all EMR countries except Somalia. In Somalia, measles surveillance changed in 2014 from case-based surveillance with laboratory testing of a limited number of cases at hospitals in two regions to aggregate reporting^§§^ of clinically compatible cases, without complete case investigations of each case, in all regions. The WHO Global Measles and Rubella Laboratory Network supports surveillance by providing laboratory confirmation and genotyping of reported cases (*7*). Measles virus genotypes are reported to the WHO global measles nucleotide surveillance database (*8*). Suspected measles cases are confirmed based on laboratory findings, an epidemiologic link, or clinical criteria. Case-based measles surveillance in EMR countries and areas is monitored using important surveillance performance indicators^¶¶^ including 1) the number of suspected measles cases ultimately discarded as nonmeasles (target = two or more per 100,000 population); 2) the proportion of second-level units (e.g., districts) with two or more discarded cases per 100,000 (target = 80%); 3) suspected cases with adequate investigation*** (target = 80%); 4) suspected cases with adequate blood specimens^†††^ (target = 80%); and 5) laboratory results available <5 days after specimen receipt (target = 80%).

During 2013–2019, the number of EMR countries and areas that met the target for suspected cases discarded as nonmeasles per 100,000 population at the national level increased from 14 (67%) to 18 (86%), and from seven (33%) to 11 (52%) at the subnational level. From 2013 to 2019, the rate of discarded nonmeasles cases decreased from 6.4 per 100,000 population to 5.4; the percentage of suspected cases with adequate investigations increased from 76% to 86%; the percentage of suspected cases with adequate specimens collected for laboratory testing decreased from 85% to 70%, and the proportion of blood specimens received by the laboratory with results available in <5 days decreased from 86% to 66% (Supplementary Table, https://stacks.cdc.gov/view/cdc/86628). The declines in the latter two performance indicators were largely because of the changes in Somalia’s surveillance and a large-scale outbreak in Yemen during 2018–2019.

## Measles Incidence and Genotypes

In EMR, reported measles cases decreased 74% from 2013 to 2014, from 16,531 to a record low of 9,499; however, in 2015, 2017, and 2018, reported measles cases increased to 21,734, 34,286 and 64,198, respectively, and then decreased to 16,703 in 2019 (Figure). Annual regional measles incidence per million population approximately tripled from 33.5 in 2013 to 91.2 in 2018, then decreased to 23.3 in 2019 (Table 1). The increase in measles cases during 2015–2018 occurred primarily because of large outbreaks in Somalia during 2015–2017, Pakistan during 2017–2018, and Yemen in 2018. The number of detected circulating measles virus genotypes in EMR decreased from four in 2013 (B3 in 13 countries, D4 in three countries, D8 in three countries, and H1 in one country) to two in 2019 (B3 in 15 countries and D8 in five countries).

**FIGURE:**
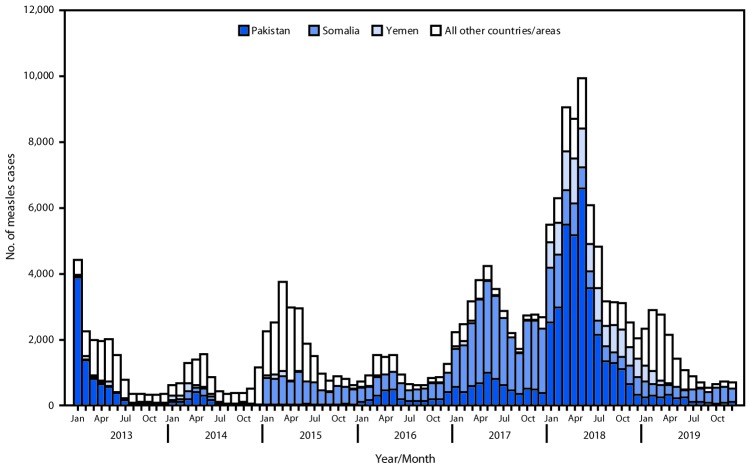
Confirmed measles cases,* by month of rash onset — World Health Organization (WHO) Eastern Mediterranean Region, 2013–2019 * Confirmed and clinically compatible measles cases reported by countries and areas to WHO. A case of measles was laboratory-confirmed when measles-specific immunoglobulin M antibody was detected in serum or measles-specific RNA was detected by polymerase chain reaction in a person who was not vaccinated during the 30 days before rash onset. A case of measles was confirmed by epidemiologic linkage when linked in time and place to a laboratory-confirmed measles case but lacked serologic confirmation. During 2013–2019, a case of measles meeting the WHO case definition but without a specimen collected could be reported as clinically compatible.

## Regional Verification of Measles Elimination

The EMR Verification Commission for Measles Elimination was established in February 2018 to evaluate the status of measles elimination in EMR countries based on documentation submitted annually by national verification committees. By the end of 2019, three (14%) EMR countries (Bahrain, Iran, and Oman) were verified as having achieved measles elimination (*9*).

## Discussion

During 2013–2018, both MCV1 and MCV2 coverage in EMR increased but remained 14 percentage points and 22 percentage points below the WHO-recommended level of ≥95%. Although a few EMR countries have achieved and maintained measles elimination, large-scale measles outbreaks in others have revealed persistent suboptimal coverage with 2 doses of MCV through routine immunization services. In several EMR countries, major challenges to implementing measles elimination activities include civil unrest, armed conflict, and unpredictable mass population displacements and resettlements that can disrupt all aspects of planning and implementation of immunization services delivery, including SIAs. Conducting SIAs in areas with no local government requires building strong partnerships and close links with local communities. Implementing periodic SIAs according to WHO SIA guidelines (https://www.who.int/immunization/diseases/measles/SIA-Field-Guide.pdf) and using the WHO SIA readiness assessment tool (http://www9.who.int/immunization/diseases/measles/en/) to ensure a high-quality activity that achieves ≥95% coverage, particularly in areas with complex humanitarian emergencies, requires the availability of adequate funds for vaccines and supplies, operational costs, and experienced personnel who can implement a complex activity in a culturally appropriate manner under challenging circumstances.

Measles elimination efforts can leverage assets, experience, and capacity from the Global Polio Eradication Initiative (GPEI). The Eastern Mediterranean Regional Technical Advisory Group on Immunization recommended forming a multipartner taskforce to apply lessons learned from the GPEI and address gaps in measles vaccination coverage. These include mapping areas where children who are missed by routine immunization services live, identifying reasons for being missed, and developing a strategic plan that includes allocation of necessary resources for implementation (*10*).

The findings in this report are subject to at least two limitations. First, administrative coverage might overestimate vaccination coverage through erroneous inclusion of SIA doses or doses administered to children outside target age groups, inaccurate estimates of the target population size, and inaccurate reports of the number of doses delivered. Second, surveillance data likely underestimate measles incidence because not all patients seek care, and not all measles cases in patients who seek care are reported.

To accelerate progress toward measles elimination in EMR, the visibility of efforts to achieve the measles elimination goal must be raised, including the benefits of achieving measles elimination. The new global guidance document to be submitted for approval by the World Health Assembly in 2020 (the Immunization Agenda 2030: A Global Strategy to Leave No One Behind [IA2030]),^§§§^ builds on lessons learned and progress made toward the Global Vaccine Action Plan goals and, importantly, identifies measles incidence as a signal for improving immunization services and strengthening primary health care systems. To achieve vaccination coverage and equity targets that leave no one behind and accelerate progress toward measles elimination and broader EMVAP and IA2030 goals, sustained and predictable investments and careful management of the leveraging of the substantial polio eradication infrastructure and resources are critically needed.

**Summary**

**What is already known about this topic?**

During 2008–2012, estimated first-dose coverage with measles-containing vaccine (MCV1) in the Eastern Mediterranean Region was 83%; reported measles cases approximately tripled, from 12,196 to 36,456, with large outbreaks in high-incidence countries.

**What is added by this report?**

Annual regional measles incidence increased from 33.5 per million population in 2013 to 91.2 in 2018, primarily because of large outbreaks in Pakistan, Somalia, and Yemen; then decreased to 23.3 in 2019.

**What are the implications for public health practice?**

To achieve measles elimination, efforts are needed to increase MCV1 and MCV2 coverage, conduct high-quality supplementary immunization activities, and reach populations at high risk for not accessing immunization services or living in areas with civil strife.

## Footnotes

* The Eastern Mediterranean Region, one of six regions of the World Health Organization, consists of 21 Member States and Palestine (West Bank and Gaza Strip), with a population of nearly 583 million persons. The member states include Afghanistan, Bahrain, Djibouti, Egypt, Iran, Iraq, Jordan, Kuwait, Lebanon, Libya, Morocco, Oman, Pakistan, Qatar, Saudi Arabia, Somalia, Sudan, Syria, Tunisia, United Arab Emirates, and Yemen.

† Measles elimination is defined as the absence of endemic measles cases for a period of ≥12 months, in the presence of adequate surveillance.

§ SIAs are immunization campaigns, typically carried out using two targeted age ranges. An initial, nationwide catch-up SIA targets all children aged 9 months–14 years, with the goal of eliminating measles susceptibility in the population. Periodic follow-up SIAs then target all children born since the last SIA. Follow-up SIAs generally are conducted every 2 to 4 years and target children aged 9–59 months; the goal of a follow-up SIA is to vaccinate children who have not received a first dose and to protect children who did not respond to the first dose of measles vaccine.

¶ Suspected cases that have been investigated and determined not to be measles using laboratory testing in a proficient laboratory or epidemiologic linkage to a laboratory-confirmed outbreak of another communicable disease that is not measles are discarded as nonmeasles cases. A proficient laboratory is one that is WHO accredited or has an established quality assurance program with oversight by a WHO accredited laboratory.

** Administrative vaccination coverage is the number of vaccine doses administered divided by the estimated target population.

†† Case-based measles surveillance includes individual case investigation and blood specimen collection for laboratory testing.

§§ Aggregate measles surveillance involves a report of a summary of suspected measles cases, by age group and location (district), but does not include a line-listing of individual cases.

¶¶ Important surveillance performance indicators include 1) two or more discarded nonmeasles cases per 100,000 population at the national level per year; 2) two or more discarded nonmeasles cases per 100,000 per year in ≥80% of subnational administrative units; 3) adequate investigation of ≥80% of suspected measles cases conducted within 48 hours of notification; 4) adequate collection and testing in a proficient laboratory of specimens from ≥80% of suspected cases for detecting acute measles and rubella infection; 5) receipt of ≥80% of specimens at the laboratory within 5 days of collection; 6) report of ≥80% of serology results by the laboratory within 4 days of specimen receipt; and 7) on-time reporting of measles and rubella data to the national level by ≥80% of surveillance units.

*** Adequate investigation is a case investigated within 48 hours of notification that includes all 10 core variables: 1) case identification; 2) date of birth/age; 3) sex; 4) place of residence; 5) vaccination status or date of last vaccination; 6) date of rash onset; 7) date of notification; 8) date of investigation; 9) date of blood sample collection; and 10) place of infection or travel history.

††† An adequate blood specimen is a sample by venipuncture in a sterile tube with a volume of 5 ml for older children and adults and 1 ml for infants and younger children. Adequate samples for antibody detection are those collected within 28 days after onset of rash.

§§§ https://www.who.int/immunization/immunization_agenda_2030/en/.
